# Racial, Ethnic, and Sex Differences in Need and Receipt of Support for Social Needs Among Veterans

**DOI:** 10.1001/jamahealthforum.2025.0992

**Published:** 2025-05-02

**Authors:** David A. Frank, Lauren E. Russell, Gregory T. Procario, Sarah M. Leder, Jennifer L. McCoy, Shane Lamba, Ernest M. Moy, Leslie R. M. Hausmann

**Affiliations:** 1 Center for Health Equity Research and Promotion (CHERP), Veterans Affairs Pittsburgh Healthcare System, Pittsburgh, Pennsylvania; 2 Office of Health Equity, Veterans Health Administration, Washington, DC; 3 Department of Medicine, University of Pittsburgh School of Medicine, Pittsburgh, Pennsylvania

## Abstract

**Importance:**

Health-related social needs, downstream manifestations of social determinants or drivers of health, impact patients’ health and well-being. To develop equity-driven social care interventions, health care systems must apply an intersectional equity lens when assessing patients’ social needs.

**Objective:**

To evaluate racial, ethnic, and sex differences in social needs and receipt of support among veterans receiving health care in the Veterans Health Administration (VHA).

**Design, Setting, and Participants:**

A cross-sectional survey study of VHA primary care patients seen in January or February 2023 was carried out in a national sample of veterans, stratified by race and ethnicity (Black, Hispanic, White), and sex (male, female). Participants were invited by mail to complete a survey online or by mail. Of those invited (N = 38 759), 7095 (18.3%) responded. Data collection occurred from March 2, 2023, through May 9, 2023. Analyses were conducted from February 15, 2024, through July 16, 2024.

**Exposures:**

Intersection of self-identified race, ethnicity, and sex.

**Main Outcomes and Measures:**

Age-adjusted prevalence ratio (aPR) of reported need for and receipt of support across 13 social need domains.

**Results:**

Analyses included 6611 respondents representing 939 467 veterans (unweighted No. of participants [weighted %]; 1089 [4.1%] Black women; 1144 [19.4%] Black men; 941 [1.6%] Hispanic women; 1281 [11.3%] Hispanic men; 805 [5.3%] White women; 1351 [58.4%] White men). After age adjustment, compared with White men, Black men had significantly higher aPRs of need for support in all domains except childcare and employment (aPRs ranged from 1.35 [95% CI, 1.09-1.69] for social isolation to 2.73 [95% CI, 1.89-3.95] for managing discrimination). Hispanic women had higher aPRs in 8 domains: childcare (aPR, 2.78; 95% CI, 1.19-6.48), discrimination (aPR, 2.69; 95% CI, 1.68-4.29), internet (aPR, 1.81; 95% CI, 1.17-2.79), housing (aPR, 1.81; 95% CI, 1.10-2.99), legal issues (aPR, 1.70; 95% CI, 1.02-2.84), loneliness (aPR, 1.67; 95% CI, 1.28-2.18), food (aPR, 1.55; 95% CI, 1.03-2.35), and social isolation (aPR, 1.40; 95% CI, 1.05-1.87). Black women had higher aPRs for discrimination (aPR, 2.68; 95% CI, 1.82-3.95), legal issues (aPR, 2.04; 95% CI, 1.40-2.97), food (aPR, 1.74; 95% CI, 1.28-2.37), loneliness (aPR, 1.60; 95% CI, 1.28-2.01), paying for basics (aPR, 1.57; 95% CI, 1.15-2.14), and social isolation (aPR, 1.48; 95% CI, 1.18-1.87). Hispanic men had higher aPRs for housing (aPR, 1.88; 95% CI, 1.18-3.02), legal issues (aPR, 1.81; 95% CI, 1.14-2.86), internet (aPR, 1.56; 95% CI, 1.13-2.16), and loneliness (aPR, 1.44; 95% CI, 1.10-1.88). White women had higher aPRs for childcare (aPR, 3.37; 95% CI, 1.36-8.35) and discrimination (aPR, 1.60; 95% CI, 1.03-2.50). There was 1 significant difference in receiving support: Black women had a lower prevalence of receiving support for work (aPR, 0.58; 95% CI, 0.35-0.94).

**Conclusions and Relevance:**

This study found that there was wide variation in the health-related social need domains in which VHA race, ethnicity, and sex subpopulations reported needing support. Applying an intersectional lens when evaluating social needs lays the groundwork for equity-guided social care interventions in the VHA.

## Key Points

**Question:**

How do intersecting racial and ethnic and sex identities impact the prevalence of need and receipt of support across 13 health-related social needs among veterans?

**Findings:**

In this cross-sectional analysis including 6611 respondents, compared with White men, Black men had significantly higher prevalence of needs in all domains except 2, Hispanic women had higher prevalence in 8 domains, Black women in 6, Hispanic men in 4, and White women in 2. Receipt of support was largely unassociated with race, ethnicity, and sex.

**Meaning:**

Need for support in health-related social need domains vary widely across race, ethnicity, and sex subpopulations of veterans.

## Teaser

This cross-sectional study examines the prevalence of self-reported social needs and receipt of support among a diverse sample of veterans receiving primary care in the veterans Health Administration.

## Introduction

Given increasing awareness of the impact of health-related social needs—the downstream manifestation of social determinants or drivers of health—on patients’ health and well-being, policymakers and professional organizations have called for health systems to improve their means of identifying and addressing these needs.^1^ A leader in the integration of medical and social care, the Veterans Health Administration (VHA) is advancing initiatives to screen for social risks and address social needs among veterans.^2,3,4,5^ Point-of-care initiatives screen patients to identify their social risks, which are specific, individual-level adverse social conditions associated with poor health.^6,7^ Positive screening for risks typically prompts a follow-up with care team members who engage patients in conversations about their social needs and provide relevant referrals or resources.^4,6,7^

Distinct from social risks, social needs reflect a person’s perceptions of need and their priorities for seeking or accepting support.^6^ Studies focused on assessing social risks in patient populations have shown steep drop offs between screening positive for a social risk, screening positive for a social need, and accepting or following through on referrals for support.^8,9,10,11,12^ A dearth of literature about patients’ endorsement of social needs, along with rapidly evolving changes in terminology and types of social health screenings being used in health care settings,^3,8^ led us to focus this study on the prevalence of self-reported social needs and receipt of support among a diverse sample of veterans receiving VHA health care.

Understanding variation in the prevalence and types of social needs experienced across demographic subpopulations served by a health care system is imperative to designing social care interventions that will promote health equity.^13,14^ Recommended methods to advance social care equity include considering how social needs differ across intersecting marginalized identities such as race, ethnicity, and sex, recognizing that race and ethnicity are social constructs and imperfect proxies for systematic disadvantage affecting minoritized racial and ethnic populations.^15^ The purpose of this study was to investigate how self-reported need for and receipt of support in health-related social domains vary by race, ethnicity, and sex in veterans receiving VHA primary care services. This study contributes new evidence regarding intersectionality in social needs among the VHA patient population and is the first study that we are aware of that examines intersectionality in social needs or receipt of support according to race, ethnicity, and sex in any large patient population.

## Methods

The Veterans Affairs Pittsburgh Healthcare System institutional review board deemed this study exempt from human subjects research oversight. We followed the Strengthening the Reporting of Observational Studies in Epidemiology (STROBE) reporting guidelines for reporting observational studies.

### Study Design and Survey Administration

We conducted a cross-sectional analysis of data collected via the VHA Your Recent Visit survey, a mechanism to assess patient experiences as part of the VHA’s Survey of Healthcare Experiences of Patients (SHEP) program.^2^ Based on the Consumer Assessment of Healthcare Providers and Systems surveys,^16^ SHEP instruments are fielded to a stratified, random sample of VHA primary care patients each month. Veterans are excluded if they are incarcerated or in hospice care, have an address outside the US, have requested to not be contacted, or have been selected to complete another SHEP survey in the previous 12 months. Eligible veterans are mailed an invitation to complete the survey online. After 1 week, they receive a reminder email and are mailed a paper survey. After 2 weeks, reminder postcards are sent. This study included veterans with VHA primary care encounters in January or February 2023. We oversampled women and Black and Hispanic veterans to reach statistical power to assess differences in social needs according to the intersection of race, ethnicity, and sex.^17,18^ Survey weights accounted for the sampling design and survey nonresponse. Data collection occurred from March 2, 2023, through May 9, 2023. Analyses were conducted from February 15, 2024, through July 16, 2024.

### Primary Exposure: Intersection of Race, Ethnicity, and Sex

The sample was designed to yield approximately 900 respondents each from 6 mutually exclusive racial and ethnic and sex (herein referenced as “race-ethnicity-sex”) groups: Black women, Black men, Hispanic women, Hispanic men, White women, and White men. For sampling purposes, age, race, ethnicity, and sex were ascertained from the VHA Corporate Data Warehouse (CDW). Race and ethnicity in the CDW are primarily based on self-reported information collected during VHA enrollment or registration for health care encounters and entered by health care staff.

Self-reported race, ethnicity, and sex were also assessed on the survey. Respondents were asked to select 1 or more races from options including American Indian or Alaska Native, Asian, Black or African American, Native Hawaiian or Other Pacific Islander, and White. Ethnicity was assessed using the question, “Are you of Hispanic or Latino origin or descent? (Yes or No).” Sex was assessed by the question, “What sex is listed on your birth certificate? (female or male).”

**Figure 1.**
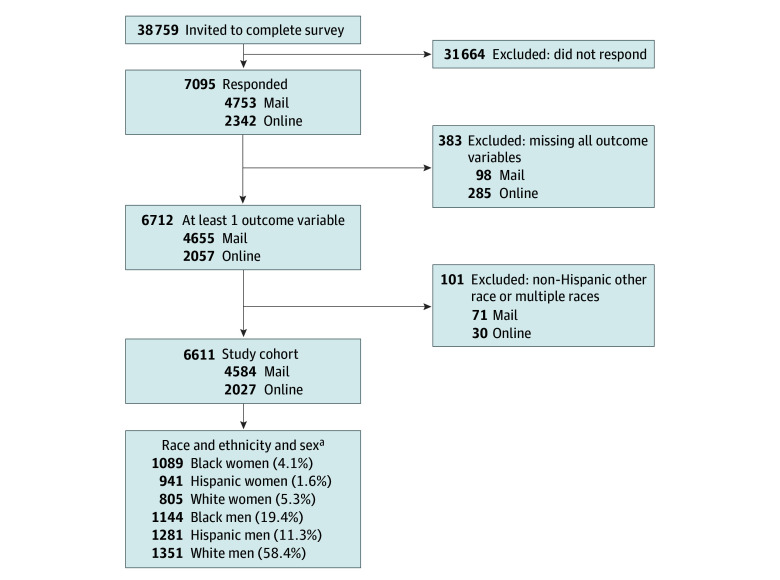
Consort Diagram for Analytic Cohort ^a^Data are expressed as unweighted number of participants and weighted percentages.

To construct race-ethnicity-sex groups, we prioritized self-reported values over administrative data. Respondents with missing self-reported race (unweighted No. of participants [weighted %]: 506 [7.7%]), ethnicity (249 [3.8%]), and/or sex (91 [1.4%]) were assigned to groups based on administrative data. Respondents who self-identified as Hispanic were categorized as Hispanic, regardless of self-reported race. Non-Hispanic Black and non-Hispanic White are subsequently referred to as Black and White, respectively. Due to small numbers, we excluded respondents who self-identified as non-Hispanic American Indian or Alaska Native, Asian, or Native Hawaiian or Other Pacific Islander, or who selected multiple races (Figure 1).

### Outcomes: Need for and Receipt of Support

The survey assessed need for support for paying for basics like food, housing, medical care, and heating; obtaining adult caregiving for yourself or others, obtaining childcare, finding or keeping work, paying for food, getting or maintaining housing, getting transportation for basic needs like medical care or grocery shopping, accessing internet at home, feeling socially isolated, feeling lonely, managing experiences of discrimination, getting assistance with legal issues, and getting additional education or job training. Participants were asked, “In the past 6 months, did you need support with any of the following?” For each domain, participants were provided 3 response options: “no support needed,” “needed support and got it,” and “needed support but did not get it.” Following the protocol for adding new items to SHEP instruments,^2^ these questions were tested through cognitive interviews with veterans (n = 8) who confirmed that the items were clear and that it was easy to select the answer that applied to their circumstances. Items were further tested by inviting members of a veteran volunteer registry (n = 3268, of which 907 [28%] responded) to complete the survey. No psychometric issues were detected, and the survey was approved for use in official postcare patient experience surveys. For analyses examining the binary outcome of needing support vs not, those who reported needing support were combined into a single category regardless of whether they reported getting support.

### Covariate and Descriptive Variables

Age group was included in adjusted models because women and racial and ethnic minoritized veterans tend to be younger than White male veterans. Age was ascertained via the survey and supplemented using administrative data when self-reported age was missing (n = 90 [1.4%]). Characteristics collected via the survey were included for descriptive purposes: education, sexual orientation, and most recent visit type (phone, video, in office).

### Statistical Analyses

We compared the prevalence of need for support, and receipt of support among those who needed it, across 6 race-ethnicity-sex groups for each domain. Weighted data were used for all analyses and weighted percentages are reported. We used Rao-Scott second-order corrected Pearson χ^2^ tests to assess differences in characteristics across groups.

We developed 2 sets of log-binomial models to examine the association between the race-ethnicity-sex groups and each outcome. We chose log-binomial models to avoid overestimating differences for common outcomes.^19^ Previous research has highlighted the importance of not overstating results when making intersectional comparisons.^18^ The first set of models estimated the prevalence of needing support for each domain. The second set of models evaluated the prevalence of receiving support for each domain among those who needed support. For all models, we performed unadjusted prevalence ratio (PR) and age-adjusted (aPR) analyses using the largest demographic group (White men) as the reference. *P* values <.05 were considered statistically significant. We did not adjust for multiple comparisons because the study was designed to detect race-ethnicity-sex differences in each social need domain and the reported analyses were planned. Data extraction, cleaning, analyses, and figure creation were performed using R statistical software (version x64 4.3.2, R Foundation).

## Results

### Sample Characteristics

Of 38 759 veterans invited, 7095 (18.3%) responded to the survey. Respondents were more likely than nonresponders to be White, male, and older (*P* < .001; eTable 1 in Supplement 1). After excluding respondents with missing outcomes and self-identified race and ethnicity other than Black, Hispanic, or White, the final sample included 6611 veterans (Figure 1).

The weighted sample represented 939 467 veterans (unweighted No. of participants [weighted %]: 1089 Black women [4.1%]; 1144 Black men [19.4%]; 941 Hispanic men [1.6%]; 1281 Hispanic men [11.3%]; 805 White women [5.3%]; 1351 White men [58.4%]; [Table 1]). Black, Hispanic, and White women tended to be younger and had higher levels of education than their male counterparts. Black and Hispanic men tended to be younger than White men. Representation of lesbian, bisexual, gay, and other (ie, “other” and “not sure”) sexual orientations varied among the groups, ranging from 3.6% among White men (n = 50) to 25.4% among Hispanic women (n = 108) (Table 1).

**Table 1.** Sociodemographic Characteristics of Survey Respondents Overall and by Race, Ethnicity, and Sex

Characteristic	Race-ethnicity-sex, No. (%)^a^							*P* value
	Overall	Women			Men			
		Black	Hispanic	White	Black	Hispanic	White	
Unweighted No. of participants	6611	1089	941	805	1144	1281	1351	NA
Weighted No. of participants	939 467	38 128	14 813	49 434	182 303	106 341	548 448	NA
Age category, y^b^								
18 to 44	596 (16.4)	127 (34.8)	242 (61.5)	77 (32.2)	23 (10.4)	93 (35.6)	34 (10.8)	<.001
45 to 54	791 (12.7)	185 (19.6)	212 (16.6)	131 (21.9)	82 (14.6)	113 (11.9)	68 (10.8)	
55 to 64	1612 (19.4)	458 (31.1)	253 (13.6)	259 (25.4)	267 (27.4)	218 (17.2)	157 (16.0)	
65 to 74	2012 (25.5)	283 (13.2)	192 (7.0)	242 (15.3)	452 (30.5)	415 (18.7)	428 (27.4)	
≥75	1600 (26.0)	36 (1.3)	42 (1.3)	96 (5.3)	320 (17.1)	442 (16.6)	664 (35.0)	
Education^c^								
High school or less	1587 (29.6)	96 (7.7)	78 (6.2)	111 (9.7)	406 (32.6)	405 (24.7)	491 (33.5)	<.001
Some college or 2-y degree	2654 (39.1)	434 (37.9)	395 (41.3)	343 (45.5)	470 (43.7)	530 (47.9)	482 (35.4)	
4-y College graduate	1088 (15.7)	233 (24.5)	228 (30.2)	140 (17.8)	128 (12.0)	170 (14.9)	189 (15.8)	
More than 4-y college graduate	1181 (15.6)	302 (29.9)	218 (22.3)	201 (27.0)	120 (11.8)	163 (12.5)	177 (15.2)	
Sexual orientation^d^								
Heterosexual	5874 (94.2)	963 (89.1)	786 (74.6)	673 (81.0)	1048 (95.5)	1158 (91.2)	1246 (96.4)	<.001
LGB, other, not sure	422 (5.8)	77 (10.9)	108 (25.4)	104 (19.0)	35 (4.5)	48 (8.8)	50 (3.6)	
Visit type^e^								
By phone	606 (8.5)	93 (8.2)	100 (10.3)	83 (10.0)	98 (8.4)	115 (8.0)	117 (8.4)	.87
Video visit	331 (5.8)	62 (6.4)	64 (7.3)	53 (7.4)	44 (5.5)	53 (7.0)	55 (5.4)	
In clinician’s office	5389 (85.7)	907 (85.4)	756 (82.5)	654 (82.5)	918 (86.1)	1045 (85.1)	1109 (86.2)	

Abbreviations: LGB, lesbian, gay, bisexual; NA, not applicable.

^a^ Data were self-reported and expressed as unweighted number of participants and weighted percentages, unless otherwise indicated.

^b^ Missing self-report data were supplemented with administrative data for 506 participants missing self-reported race, 249 missing self-reported ethnicity, 91 missing self-reported sex, and 90 missing self-reported age.

^c^ Excludes 101 participants for whom data on education were missing.

^d^ Excludes 315 participants for whom data on sexual orientation were missing.

^e^ Excludes 285 participants for whom data on visit type were missing. Based on the visit type associated with the primary care visit between January and February 2023 connected with this survey.

Unadjusted prevalence of need for support ranged from 2.5% for obtaining childcare to 28.5% for feeling lonely and varied across race-ethnicity-sex subpopulations (Table 2). Hispanic women had the highest unadjusted rate of needing support, followed by Black women, for 7 domains. Black and Hispanic men tended to report needing support more than White women, and White men reported the lowest unadjusted need for support among all groups for 11 of 13 domains (Table 2).

**Table 2.** Weighted Need for and Receipt of Support for Social Domains, Overall and by Self-Reported Race, Ethnicity, and Sex

Characteristic	Race-ethnicity-sex, weighted %						
	Overall	Black women	Hispanic women	White women	Black men	Hispanic men	White men
Unweighted No. of participants	6611	1089	941	805	1144	1281	1351
Weighted No. of participants	939 467	38 128	14 813	49 434	182 303	106 341	548 448
Feeling lonely							
No support needed	71.5	52.0	45.0	63.8	63.2	61.2	79.0
Needed support	28.5	48.0	55.0	36.2	36.8	38.8	21.0
Got support	56.7	52.7	51.3	57.6	59.2	60.3	55.0
Did not get support	43.3	47.3	48.7	42.4	40.8	39.7	45.0
Feeling socially isolated							
No support needed	74.3	55.1	53.2	68.3	68.2	67.4	80.2
Needed support	25.7	44.9	46.8	31.7	31.8	32.6	19.8
Got support	56.3	52.6	58.3	50.4	59.2	58.5	55.3
Did not get support	43.7	47.4	41.7	49.6	40.8	41.5	44.7
Paying for basics							
No support needed	81.7	70.5	70.8	80.3	74.3	76.6	86.2
Needed support	18.3	29.5	29.2	19.7	25.7	23.4	13.8
Got support	52.2	43.6	47.7	48.6	50.4	59.3	53.0
Did not get support	47.8	56.4	52.3	51.4	49.6	40.7	47.0
Paying for food							
No support needed	84.0	71.8	73.6	80.3	77.6	79.8	88.4
Needed support	16.0	28.2	26.4	19.7	22.4	20.2	11.6
Got support	57.3	50.8	63.1	51.1	57.5	60.6	57.7
Did not get support	42.7	49.2	36.9	48.9	42.5	39.4	42.3
Accessing the internet at home							
No support needed	86.8	85.3	82.6	92.2	81.2	83.5	89.0
Needed support	13.2	14.7	17.4	7.8	18.8	16.5	11.0
Got support	58.2	59.7	58.1	62.9	56.4	55.3	59.7
Did not get support	41.8	40.3	41.9	37.1	43.6	44.7	40.3
Assistance with legal issues							
No support needed	87.4	79.8	83.0	88.4	78.2	83.4	91.7
Needed support	12.6	20.2	17.0	11.6	21.8	16.6	8.3
Got support	41.4	37.1	29.4	35.9	43.6	29.0	46.5
Did not get support	58.6	62.9	70.6	64.1	56.4	71.0	53.5
Transportation for basic needs							
No support needed	88.5	85.5	82.7	88.4	82.5	86.0	91.3
Needed support	11.5	14.5	17.3	11.6	17.5	14.0	8.7
Got support	67.5	64.8	60.1	61.2	65.8	69.0	69.6
Did not get support	32.5	35.2	39.9	38.8	34.2	31.0	30.4
Adult caregiving for self or others							
No support needed	88.7	87.4	87.9	90.5	84.2	88.1	90.2
Needed support	11.3	12.6	12.1	9.5	15.8	11.9	9.8
Got support	62.3	57.3	53.3	60.5	62.3	63.6	63.0
Did not get support	37.7	42.7	46.7	39.5	37.7	36.4	37.0
Managing experiences of discrimination							
No support needed	88.7	74.6	73.7	85.4	79.2	87.5	93.7
Needed support	11.3	25.4	26.3	14.6	20.8	12.5	6.3
Got support	36.5	32.3	38.3	33.5	42.8	37.2	31.0
Did not get support	63.5	67.7	61.7	66.5	57.2	62.8	69.0
Getting additional education or job training							
No support needed	88.9	79.4	77.8	87.8	83.3	81.9	93.2
Needed support	11.1	20.6	22.2	12.2	16.7	18.1	6.8
Got support	38.9	34.7	46.1	49.2	40.2	29.8	41.1
Did not get support	61.1	65.3	53.9	50.8	59.8	70.2	58.9
Getting or maintaining housing							
No support needed	89.8	87.4	81.2	91.5	82.8	84.0	93.5
Needed support	10.2	12.6	18.8	8.5	17.2	16.0	6.5
Got support	50.4	45.2	68.6	38.0	52.3	41.9	53.5
Did not get support	49.6	54.8	31.4	62.0	47.7	58.1	46.5
Finding or keeping work							
No support needed	90.8	83.9	79.0	86.7	89.2	86.2	93.4
Needed support	9.2	16.1	21.0	13.3	10.8	13.8	6.6
Got support	41.9	34.8	54.2	49.0	42.2	31.8	44.6
Did not get support	58.1	65.2	45.8	51.0	57.8	68.2	55.4
Obtaining childcare							
No support needed	97.5	94.9	91.8	92.8	97.6	95.2	98.7
Needed support	2.5	5.1	8.2	7.2	2.4	4.8	1.3
Got support	39.1	29.8	59.2	46.1	55.1	36.7	26.8
Did not get support	60.9	70.2	40.8	53.9	44.9	63.3	73.2

Among those reporting a need for support, the unadjusted percentage of respondents who needed support but did not get it ranged from 32.5% for transportation to 63.5% for managing experiences of discrimination (Table 2). Across all domains, Black women more often reported not getting support when it was needed compared with other groups. Hispanic women, Hispanic men, and White women tended to have similar rates of not getting support when it was needed. Black and White men tended to have the lowest unadjusted rates of not getting support when needed (Table 2).

### Age-Adjusted Differences in Prevalence of Need for and Receipt of Support Across Race, Ethnicity, and Sex

**Figure 2.**
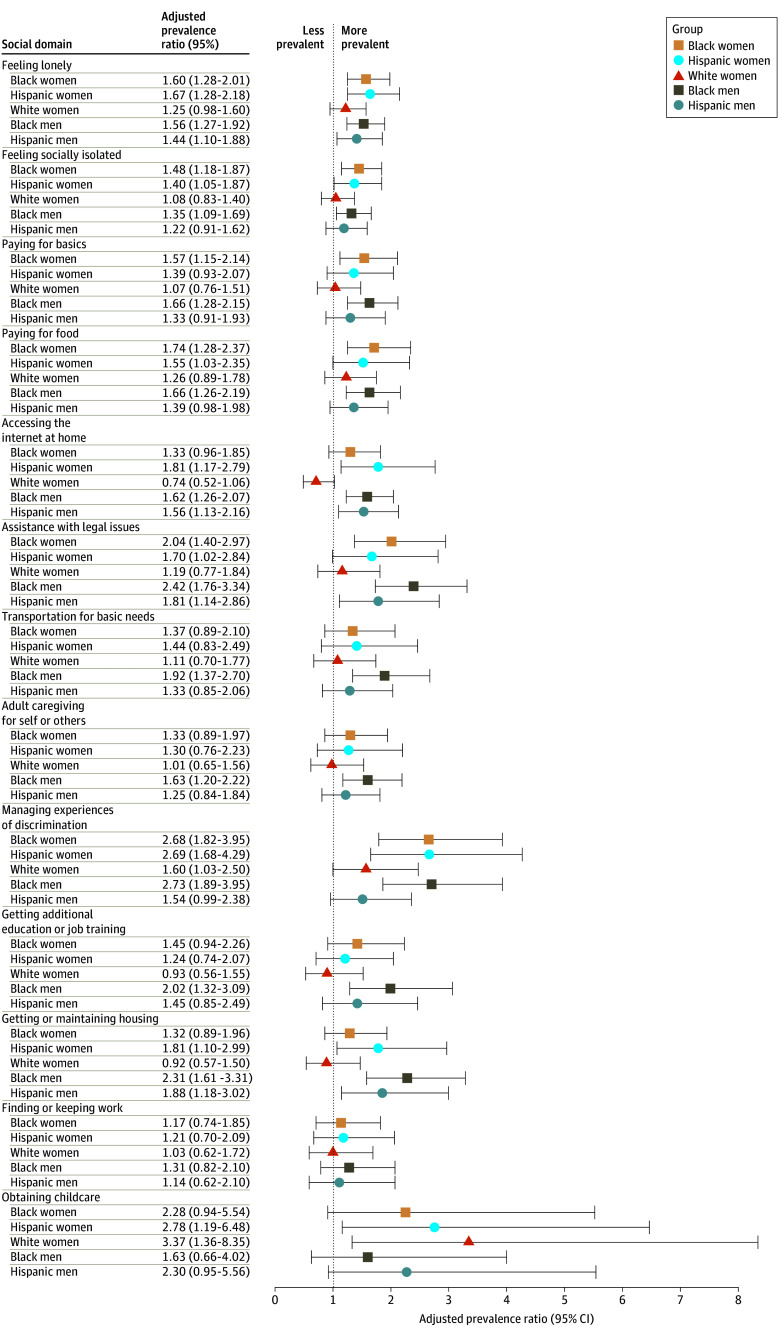
Race-Ethnicity-Sex Differences in Age-Adjusted Prevalence of Needing Support in Social Domains All models were adjusted for age groups. Adjusted prevalence ratios greater than 1 indicate that the race-ethnicity-sex group had a higher prevalence of endorsed need for a particular social domain compared with the reference group of White men.

Age-adjusted models using White men as the reference group showed at least 1 significant race-ethnicity-sex difference in need for support (vs not) for all domains except finding or keeping work (Figure 2; eTable 2 in Supplement 1). Compared with White men, aPRs of needing support for experiences of discrimination were significantly higher across all race-ethnicity-sex groups except for Hispanic men, with significant aPRs ranging from 1.60 (95% CI, 1.03-2.50) among White women to 2.69 (95% CI, 1.68-4.29) for Hispanic women, the latter indicating that the need for support for discrimination was 2.69 times more prevalent among Hispanic women than among White men. For feeling lonely and help with legal issues, aPRs of needing support were significantly higher for all race-ethnicity-sex groups other than White women (Figure 2; eTable 2 in Supplement 1). For accessing internet at home and obtaining or keeping housing, aPRs were higher for Hispanic women, Hispanic men, and Black men (Figure 2; eTable 2 in Supplement 1). For social isolation and paying for food, aPRs were higher for Hispanic women, Black women, and Black men (Figure 2; eTable 2 in Supplement 1). Need for support with obtaining childcare was significantly higher only among Hispanic women (aPR, 2.78; 95% CI, 1.19-6.48) and White women (aPR, 3.37; 95% CI, 1.36-8.35).

Age-adjusted need for support in some domains was significantly higher only among Black respondents. Specifically, Black women (aPR, 1.57; 95% CI, 1.15-2.14) and Black men (aPR, 1.66; 95% CI, 1.28-2.15) were more likely than White men to report needing support with paying for basics. Black men also had a significantly higher prevalence of needing support for transportation (aPR, 1.92; 95% CI, 1.37-2.70), adult caregiving (aPR, 1.63; 95% CI, 1.20-2.22), and education (aPR, 2.02; 95% CI, 1.32-3.09).

**Figure 3.**
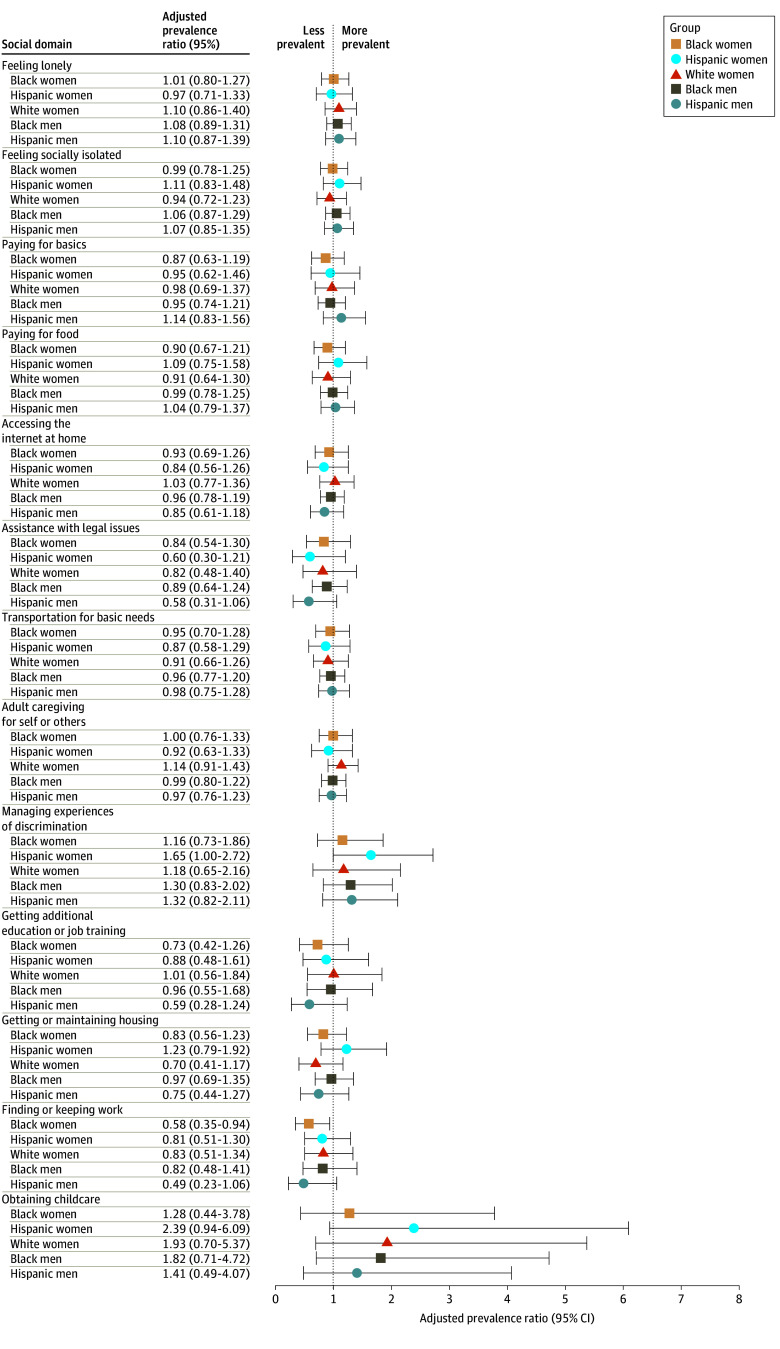
Race-Ethnicity-Sex Differences in Age-Adjusted Prevalence of Getting Support Among Those Reporting Need All models were adjusted for age groups. Adjusted prevalence ratios greater than 1 indicate that the race-ethnicity-sex group had a higher prevalence of getting support for an endorsed need for a particular social domain compared with the reference group of White men.

The second set of models examining age-adjusted differences in prevalence of getting support when it was needed showed 1 race-ethnicity-sex difference (Figure 3; eTable 3 in Supplement 1). Specifically, compared with White men, Black women had a significantly lower prevalence of receiving support for finding or keeping work (aPR, 0.58; 95% CI, 0.35-0.94).

## Discussion

This cross-sectional study provides detailed information regarding intersectional differences in social needs among VHA patients. Prior studies examining prevalence and impact of social risks or needs among veterans have primarily included older, White male veterans, the largest veteran demographic group, and have not been designed to test intersectional race-ethnicity-sex comparisons.^20,21,22,23,24,25,26,27,28^ Our focus on intersectional variation in need is appropriate given that percentages of women, individuals from minoritized racial groups, and Hispanic individuals in the veteran population are projected to rise to 18.5%, 28.0%, and 15.3%, respectively, by 2050.^29,30^

These findings demonstrated substantial variation in age-adjusted need for support among Black, Hispanic, and White male and female VHA patients. Compared with White men, Black men had a higher prevalence of need for support in all but 2 domains (finding work and childcare). Hispanic women had a higher prevalence of need in 8 domains (discrimination, feeling lonely, legal issues, internet, housing, social isolation, food, and childcare), Black women in 6 (discrimination, feeling lonely, legal issues, social isolation, food, and paying for basics), Hispanic men in 4 (feeling lonely, help with legal issues, internet, and housing), and White women in 2 (discrimination and childcare).

The patterns of need observed across groups can inform VHA efforts to identify and address health-related social needs in several ways. First, although universal social risk screening and consistent provision of support for social needs are long-term goals in the VHA,^3,4,5^ our findings suggest that some subgroups, specifically Black men, Black women, and Hispanic women, may benefit from screening more than others because they are more likely to have needs. To prevent screening initiatives from exacerbating existing disparities, it is important to ensure that screening efforts reach these subpopulations and that resources offered to those in need are thoughtfully and appropriately tailored. Second, need for support for legal issues was higher among Black and Hispanic men and women than among White men. The racialized nature of legal needs among veterans should be acknowledged and considered when the VHA establishes medical-legal partnerships, seeking out practitioners who can provide race-conscious legal aid.^31^ Third, with the exception of Hispanic men, all other race-ethnicity-sex groups reported a higher prevalence of need for managing experiences of discrimination compared with White men. Although discrimination is a well-established social driver of health,^32,33,34^ experiences of discrimination are rarely measured or addressed in health care settings. Supporting those who are subjected to discrimination as a chronic stressor is important, not only because of discrimination’s direct negative effects on health, but also because it can exacerbate other social needs including loneliness and social isolation.^35^ Moving forward, social care interventions that aim to address inequities in health should include ways to identify and support those who need help managing discrimination. Promoting the use of effective race and ethnicity–based trauma interventions^36,37,38,39,40,41^ and including discrimination as a domain in social needs screeners are steps health care systems can take to address the negative health effects of discrimination.

Finally, we observed almost no age-adjusted differences in prevalence of getting support (vs not) among those who needed it, suggesting that the likelihood of getting support when it is needed is largely unaffected by veterans’ race, ethnicity, or sex. Because we did not assess the sources of support respondents received (eg, family, friends, religious communities, public services, or Department of Veterans Affairs benefits) or whether support was adequate, future studies should examine preferences for and effectiveness of different types of support across race-ethnicity-sex groups. It is also important to note that existing literature demonstrates that some demographic characteristics, including minoritized race and ethnicity, are associated with higher self-reported need for support, with or without a corresponding social risk,^10^ and higher concordance between screening positive for a social risk and the desire for support.^9,42^ Future research should continue to examine how race, ethnicity, and sex influence self-reported social risks and needs, types of support received, and whether support received is adequate.

### Limitations

This cross-sectional study has limitations. Generalizability is limited to VHA primary care patients from the target race-ethnicity-sex groups who are likely to respond to surveys. The reliance on self-reported data, although patient-centered, also makes the findings susceptible to social desirability and recall biases. We acknowledge that, although the social needs survey used in this study went through systematic internal testing prior to being fielded, this is the first large-scale use of this survey and further item testing may be warranted. We did not assess what types of support respondents received nor whether the support adequately met the need. Finally, we were unable to assess all forms of intersectionality that may be relevant to the VHA patient population. Despite these limitations, our findings have important implications for understanding racial, ethnic, and sex differences in reported social needs and receipt of support for these needs among veterans.

## Conclusions

This study found important racial and ethnic and sex differences in the prevalence of need for support across patients receiving care from the VHA, the largest integrated health care system in the US. Understanding the intersectionality in need for support across different domains is a crucial step toward developing equitable interventions that are centered around the specific social needs of marginalized individuals.^13,14^ This intersectional analysis offers a model for health care systems seeking to develop equity-driven social care screening practices and interventions.

## Notes

Supplement 1. eTable 1. Comparison of Survey Responders and Non-Responders eTable 2. Race-Ethnicity-Sex Differences in Prevalence and Age-Adjusted Prevalence of Needing Support for Social Domains eTable 3. Race-Ethnicity-Sex Differences in Prevalence and Age-Adjusted Prevalence of Getting Support Among Those Reporting Need

Supplement 2. Data Sharing Statement

## References

[1] Institute of Medicine. Capturing Social and Behavioral Domains in Electronic Health Records: Phase 1. National Academies Press (US); 2014. Accessed May 22, 2024. https://www.ncbi.nlm.nih.gov/books/NBK195994/24757748

[2] Hausmann LRM, Cohen AJ, Eliacin J, . Developing a brief assessment of social risks for the Veterans Health Administration Survey of Healthcare Experiences of Patients. Health Serv Res. 2023;58(6):1209-1223. doi:10.1111/1475-6773.14220 37674359 PMC10622278

[3] Cohen AJ, Russell LE, Elwy AR, . Adaptation of a social risk screening and referral initiative across clinical populations, settings, and contexts in the Department of Veterans Affairs Health System. Front Health Serv. 2023;2:958969. doi:10.3389/frhs.2022.958969 36925883 PMC10012714

[4] Russell LE, Cohen AJ, Chrzas S, . Implementing a Social Needs Screening and Referral Program Among Veterans: Assessing Circumstances & Offering Resources for Needs (ACORN). J Gen Intern Med. 2023;38(13):2906-2913. doi:10.1007/s11606-023-08181-9 37165261 PMC10171907

[5] List JM, Russell LE, Hausmann LRM, . Addressing veteran health-related social needs: how joint commission standards accelerated integration and expansion of tools and services in the Veterans Health Administration. Jt Comm J Qual Patient Saf. 2024;50(1):34-40. doi:10.1016/j.jcjq.2023.10.002 37923670

[6] Russell LE, Cornell PY, Halladay CW, . Sociodemographic and clinical characteristics associated with veterans’ digital needs. JAMA Netw Open. 2024;7(11):e2445327. doi:10.1001/jamanetworkopen.2024.45327 39546310 PMC11568462

[7] Russell LE, Mitchell KM, Kennedy MA, . Building tailored resource guides to address social risks and advance health equity in the Veterans Health Administration. Fed Pract. 2024;41(1):22-28. doi:10.12788/fp.0446 38835360 PMC11147443

[8] Alderwick H, Gottlieb LM. Meanings and misunderstandings: a social determinants of health lexicon for health care systems. Milbank Q. 2019;97(2):407-419. doi:10.1111/1468-0009.12390 31069864 PMC6554506

[9] Tuzzio L, Wellman RD, De Marchis EH, . Social risk factors and desire for assistance among patients receiving subsidized health care insurance in a US-based integrated delivery system. Ann Fam Med. 2022;20(2):137-144. doi:10.1370/afm.2774 35346929 PMC8959745

[10] Schiavoni KH, Helscel K, Vogeli C, . Prevalence of social risk factors and social needs in a Medicaid Accountable Care Organization (ACO). BMC Health Serv Res. 2022;22(1):1375. doi:10.1186/s12913-022-08721-9 36403024 PMC9675191

[11] De Marchis EH, Hessler D, Fichtenberg C, . Assessment of social risk factors and interest in receiving health care-based social assistance among adult patients and adult caregivers of pediatric patients. JAMA Netw Open. 2020;3(10):e2021201. doi:10.1001/jamanetworkopen.2020.21201 33064137 PMC7568201

[12] De Marchis EH, Alderwick H, Gottlieb LM. Do patients want help addressing social risks? J Am Board Fam Med. 2020;33(2):170-175. doi:10.3122/jabfm.2020.02.190309 32179597

[13] Peek ME, Gottlieb LM, Doubeni CA, . Advancing health equity through social care interventions. Health Serv Res. 2023;58(Suppl 3)(suppl 3):318-326. doi:10.1111/1475-6773.14244 38015863 PMC10684037

[14] Cené CW, Viswanathan M, Fichtenberg CM, . Racial health equity and social needs interventions: a review of a scoping review. JAMA Netw Open. 2023;6(1):e2250654. doi:10.1001/jamanetworkopen.2022.50654 36656582 PMC9857687

[15] Flanagin A, Frey T, Christiansen SL; AMA Manual of Style Committee. Updated guidance on the reporting of race and ethnicity in medical and science journals. JAMA. 2021;326(7):621-627. doi:10.1001/jama.2021.13304 34402850

[16] Center for Medicare and Medicaid Services. Consumer Assessment of Healthcare Providers & Systems (CAHPS) | CMS. Accessed August 5, 2024. www.cms.gov/data-research/research/consumer-assessment-healthcare-providers-systems

[17] Bowleg L. The problem with the phrase women and minorities: intersectionality-an important theoretical framework for public health. Am J Public Health. 2012;102(7):1267-1273. doi:10.2105/AJPH.2012.300750 22594719 PMC3477987

[18] Bauer GR. Incorporating intersectionality theory into population health research methodology: challenges and the potential to advance health equity. Soc Sci Med. 2014;110:10-17. doi:10.1016/j.socscimed.2014.03.022 24704889

[19] Barros AJ, Hirakata VN. Alternatives for logistic regression in cross-sectional studies: an empirical comparison of models that directly estimate the prevalence ratio. BMC Med Res Methodol. 2003;3(1):21. doi:10.1186/1471-2288-3-21 14567763 PMC521200

[20] Blosnich JR, Montgomery AE, Taylor LD, Dichter ME. Adverse social factors and all-cause mortality among male and female patients receiving care in the Veterans Health Administration. Prev Med. 2020;141:106272. doi:10.1016/j.ypmed.2020.106272 33022319

[21] Vogt D, Borowski S, Maguen S, . Strengths and vulnerabilities: comparing post-9/11 U.S. veterans’ and non-veterans’ perceptions of health and broader well-being. SSM Popul Health. 2022;19:101201. doi:10.1016/j.ssmph.2022.101201 36046065 PMC9421326

[22] Gurewich D, Shoushtari SI, Ostrow R, . Prevalence and determinants of unmet social needs among rural and urban veterans. J Health Care Poor Underserved. 2023;34(1):275-292. doi:10.1353/hpu.2023.0018 37464494

[23] Kamdar N, Khan S, Brostow DP, . Association between modifiable social determinants and mental health among post-9/11 Veterans: a systematic review. J Mil Veteran Fam Health. 2023;9(3):8-26. doi:10.3138/jmvfh-2022-0025 37886122 PMC10601397

[24] Davis CI, Montgomery AE, Dichter ME, Taylor LD, Blosnich JR. Social determinants and emergency department utilization: findings from the Veterans Health Administration. Am J Emerg Med. 2020;38(9):1904-1909. doi:10.1016/j.ajem.2020.05.078 32739860

[25] Rao M, Greene L, Nelson K, Maciejewski ML, Zulman DM. Associations between social risks and primary care utilization among medically complex veterans. J Gen Intern Med. 2023;38(15):3339-3347. doi:10.1007/s11606-023-08269-2 37369890 PMC10682359

[26] Cole MB, Nguyen KH. Unmet social needs among low-income adults in the United States: associations with health care access and quality. Health Serv Res. 2020;55(Suppl 2)(suppl 2):873-882. doi:10.1111/1475-6773.13555 32880945 PMC7518813

[27] Crone B, Metraux S, Sbrocco T. Health service access among homeless veterans: health access challenges faced by homeless African American veterans. J Racial Ethn Health Disparities. 2022;9(5):1828-1844. doi:10.1007/s40615-021-01119-z 34402040 PMC8367031

[28] Flike K, Byrne T. Systematic review of access to healthcare and social services among US women veterans experiencing homelessness. Womens Health (Lond). 2023;19:17455057231189550. doi:10.1177/17455057231189550 37522527 PMC10392165

[29] National Center for Veterans Analysis and Statistics. Table 1L: VETPOP2020 living veterans by age group, gender, 2020-2050. Accessed August 5, 2024. https://www.va.gov/vetdata/docs/Demographics/New_Vetpop_Model/1L_VetPop2020_National_NCVAS.xlsx

[30] National Center for Veterans Analysis and Statistics. Table 3L: VETPOP2020 living veterans by race/ethnicity, gender, 2020-2050. Accessed August 5, 2024. https://www.va.gov/vetdata/docs/Demographics/New_Vetpop_Model/3L_VetPop2020_Race_Ethnicity_National_NCVAS.xlsx

[31] Tsai J, Middleton M, Retkin R, . Partnerships between health care and legal providers in the Veterans Health Administration. Psychiatr Serv. 2017;68(4):321-323. doi:10.1176/appi.ps.201600486 27903138

[32] Williams DR, Lawrence JA, Davis BA, Vu C. Understanding how discrimination can affect health. Health Serv Res. 2019;54(Suppl 2)(suppl 2):1374-1388. doi:10.1111/1475-6773.13222 31663121 PMC6864381

[33] Paradies Y, Ben J, Denson N, . Racism as a determinant of health: a systematic review and meta-analysis. PLoS One. 2015;10(9):e0138511. doi:10.1371/journal.pone.0138511 26398658 PMC4580597

[34] Pascoe EA, Smart Richman L. Perceived discrimination and health: a meta-analytic review. Psychol Bull. 2009;135(4):531-554. doi:10.1037/a0016059 19586161 PMC2747726

[35] Ransome Y, Valido AD, Espelage DL, . A systematic review of how social connectedness influences associations between racism and discrimination on health outcomes. Epidemiol Rev. 2023;45(1):44-62. doi:10.1093/epirev/mxad009 37477041 PMC10748800

[36] Wang C, Malaktaris A, McLean CL, . Mitigating the health effects of systemic racism: evaluation of the Race-Based Stress and Trauma Empowerment intervention. Contemp Clin Trials. 2023;127:107118. doi:10.1016/j.cct.2023.107118 36796623 PMC10389054

[37] Carlson M, Endlsey M, Motley D, Shawahin L, Williams M. Addressing the impact of racism on veterans of color: a race-based stress and trauma intervention. Psychol Violence. 2018;8(6):748-762. doi:10.1037/vio0000221

[38] Conway-Phillips R, Dagadu H, Motley D, . Qualitative evidence for Resilience, Stress, and Ethnicity (RiSE): a program to address race-based stress among Black women at risk for cardiovascular disease. Complement Ther Med. 2020;48:102277. doi:10.1016/j.ctim.2019.102277 31987226

[39] Saban KL, Motley D, Shawahin L, . Preliminary evidence for a race-based stress reduction intervention for Black women at risk for cardiovascular disease. Complement Ther Med. 2021;58:102710. doi:10.1016/j.ctim.2021.102710 33727090

[40] Taylor JY, Jones-Patten A, Prescott L, . The race-based stress reduction intervention (RiSE) study on African American women in NYC and Chicago: design and methods for complex genomic analysis. PLoS One. 2024;19(4):e0295293. doi:10.1371/journal.pone.0295293 38598554 PMC11006145

[41] Hall-Clark BN, Zabelski S, Montanaro E, Besse S, Cramer RJ. Adapting trauma-informed interventions for racial trauma considerations: a training model. Pract Innov (Wash D C). 2024;9(1):39-50. doi:10.1037/pri0000222

[42] Gruß I, Varga A, Brooks N, Gold R, Banegas MP. Patient interest in receiving assistance with self-reported social risks. J Am Board Fam Med. 2021;34(5):914-924. doi:10.3122/jabfm.2021.05.210069 34535517

