# Omission in Discussion Section

**DOI:** 10.1001/jamahealthforum.2025.2426

**Published:** 2025-06-13

The Original Investigation titled “Racial, Ethnic, and Sex Differences in Need and Receipt of Support for Social Needs Among Veterans,”^1^ published on May 2, 2025, was corrected to add a missing sentence to the second paragraph of the Discussion section. This article was corrected online.
