# Supplementary material for: Racial, Ethnic, and Sex Differences in Need and Receipt of Support for Social Needs Among Veterans
Source: JAMA Health Forum. 2025 May 2;6(5):e250992. doi: 10.1001/jamahealthforum.2025.0992 (PMC12048852; doi:10.1001/jamahealthforum.2025.0992)
Supplement: Supplement 1. — eTable 1. Comparison of Survey Responders and Non-Responders eTable 2. Race-Ethnicity-Sex Differences in Prevalence and Age-Adjusted Prevalence of Needing Support for Social Domains eTable 3. Race-Ethnicity-Sex Differences in Prevalence and Age-Adjusted Prevalence of Getting Support Among Those Reporting Need [file jamahealthforum-e250992-s001.pdf]

Supplemental Online Content

Frank DA, Russell LE, Procario GT, et al. Racial, ethnic, and sex differences in need and receipt of support for social needs among veterans. JAMA *Health Forum*. 2025;6(5):e250992. doi:10.1001/jamahealthforum.2025.0992

eTable 1. Comparison of Survey Responders and Non-Responders

eTable 2. Race-Ethnicity-Sex Differences in Prevalence and Age-Adjusted Prevalence of Needing Support for Social Domains

eTable 3. Race-Ethnicity-Sex Differences in Prevalence and Age-Adjusted Prevalence of Getting Support Among Those Reporting Need

This supplemental material has been provided by the authors to give readers additional information about their work.

© 2025 Frank DA et al. *JAMA Health Forum*.

eTable 1: Comparison of Survey Responders and Non-Responders[a]

**Characteristic**	**Survey Recipients**		
	**Non-Responder**	**Responder**	**P value**
No. of participants	31,664	7,095	
Sampled strata			
Black Female	7801 (24.6)	1199 (16.9)	
Hispanic Female	7850 (24.8)	1059 (14.9)	<0.001
White Female	3549 (11.2)	851 (12.0)	
Black Male	4778 (15.1)	1222 (17.2)	
Hispanic Male	4660 (14.7)	1390 (19.6)	
White Male	3026 (9.6)	1374 (19.4)	
Age group			
18 - 34	4785 (15.1)	161 (2.3)	<0.001
35 - 44	7105 (22.4)	546 (7.7)	
45 - 54	6319 (20.0)	901 (12.7)	
55 - 64	6459 (20.4)	1736 (24.5)	
65 to 74	4669 (14.8)	2121 (29.9)	
75 or older	2327 (7.4)	1630 (23.0)	
Type of visit[b]			
By phone	5659 (17.9)	1171 (16.5)	<0.001
Video visit	2630 (8.3)	429 (6.1)	
In clinician's office	23365 (73.8)	5488 (77.4)	

[a] Data were extracted from the administrative record and are expressed as No.(%) unless otherwise indicated.
[b] Based on the visit type associated with the primary care visit between January and February 2023 connected with this survey.

© 2025 Frank DA et al. *JAMA Health Forum*.

eTable 2: Race-Ethnicity-Sex Differences in Prevalence and Age-Adjusted Prevalence of Needing Support for Social Domains

	Unadjusted		Adjusted[a]	
	Prevalence ratio [95% CI]	P-value	Prevalence ratio [95% CI]	P-value
Feeling lonely				
Black Female	2.28 [1.91 to 2.72]	<0.001	1.60 [1.28 to 2.01]	<0.001
Hispanic Female	2.62 [2.15 to 3.18]	<0.001	1.67 [1.28 to 2.18]	<0.001
White Female	1.72 [1.40 to 2.12]	<0.001	1.25 [0.98 to 1.60]	0.08
Black Male	1.75 [1.45 to 2.11]	<0.001	1.56 [1.27 to 1.92]	<0.001
Hispanic Male	1.84 [1.46 to 2.32]	<0.001	1.44 [1.10 to 1.88]	0.007
White Male	1 [Reference]		1 [Reference]	
18 to 44			2.01 [1.57 to 2.57]	<0.001
45 to 54			1.86 [1.51 to 2.29]	<0.001
55 to 64			1.40 [1.17 to 1.68]	<0.001
65 to 74			1 [Reference]	
75 or older			0.74 [0.60 to 0.91]	0.004
Feeling socially isolated				
Black Female	2.27 [1.88 to 2.74]	<0.001	1.48 [1.18 to 1.87]	<0.001
Hispanic Female	2.37 [1.90 to 2.94]	<0.001	1.40 [1.05 to 1.87]	0.02
White Female	1.61 [1.28 to 2.01]	<0.001	1.08 [0.83 to 1.40]	0.56
Black Male	1.61 [1.32 to 1.97]	<0.001	1.35 [1.09 to 1.69]	0.007
Hispanic Male	1.65 [1.30 to 2.09]	<0.001	1.22 [0.91 to 1.62]	0.18
White Male	1 [Reference]		1 [Reference]	
18 to 44			2.24 [1.70 to 2.95]	<0.001
45 to 54			2.15 [1.72 to 2.70]	<0.001
55 to 64			1.65 [1.35 to 2.02]	<0.001
65 to 74			1 [Reference]	
75 or older			0.64 [0.51 to 0.80]	<0.001
Paying for basics				
Black Female	2.14 [1.69 to 2.72]	<0.001	1.57 [1.15 to 2.14]	0.004
Hispanic Female	2.12 [1.58 to 2.85]	<0.001	1.39 [0.93 to 2.07]	0.11
White Female	1.43 [1.07 to 1.91]	0.02	1.07 [0.76 to 1.51]	0.71
Black Male	1.87 [1.47 to 2.38]	<0.001	1.66 [1.28 to 2.15]	<0.001
Hispanic Male	1.70 [1.22 to 2.38]	0.002	1.33 [0.91 to 1.93]	0.14
White Male	1 [Reference]		1 [Reference]	
18 to 44			1.58 [1.12 to 2.25]	0.01
45 to 54			1.18 [0.88 to 1.57]	0.26
55 to 64			1.01 [0.81 to 1.26]	0.93
65 to 74			1 [Reference]	
75 or older			0.51 [0.40 to 0.66]	<0.001
Paying for food				
Black Female	2.44 [1.90 to 3.13]	<0.001	1.74 [1.28 to 2.37]	<0.001

© 2025 Frank DA et al. *JAMA Health Forum*.

	Unadjusted		Adjusted[a]	
	**Prevalence ratio [95% CI]**	**P-value**	**Prevalence ratio [95% CI]**	**P-value**
Paying for food (continued)				
Hispanic Female	2.28 [1.66 to 3.15]	<0.001	1.55 [1.03 to 2.35]	0.04
White Female	1.70 [1.25 to 2.32]	<0.001	1.26 [0.89 to 1.78]	0.20
Black Male	1.94 [1.49 to 2.52]	<0.001	1.66 [1.26 to 2.19]	<0.001
Hispanic Male	1.75 [1.29 to 2.36]	<0.001	1.39 [0.98 to 1.98]	0.06
White Male	1 [Reference]		1 [Reference]	
18 to 44			1.46 [1.00 to 2.13]	0.05
45 to 54			1.41 [1.05 to 1.91]	0.02
55 to 64			1.26 [0.99 to 1.60]	0.06
65 to 74			1 [Reference]	
75 or older			0.45 [0.33 to 0.59]	<0.001
Accessing the internet at home				
Black Female	1.34 [1.02 to 1.76]	0.04	1.33 [0.96 to 1.85]	0.09
Hispanic Female	1.58 [1.13 to 2.21]	0.008	1.81 [1.17 to 2.79]	0.008
White Female	0.71 [0.51 to 0.98]	0.04	0.74 [0.52 to 1.06]	0.10
Black Male	1.71 [1.35 to 2.17]	<0.001	1.62 [1.26 to 2.07]	<0.001
Hispanic Male	1.50 [1.10 to 2.04]	0.01	1.56 [1.13 to 2.16]	0.007
White Male	1 [Reference]		1 [Reference]	
18 to 44			0.68 [0.41 to 1.13]	0.13
45 to 54			0.54 [0.37 to 0.81]	0.003
55 to 64			1.31 [1.01 to 1.70]	0.04
65 to 74			1 [Reference]	
75 or older			0.82 [0.64 to 1.06]	0.12
Assistance with legal issues				
Black Female	2.45 [1.82 to 3.28]	<0.001	2.04 [1.40 to 2.97]	<0.001
Hispanic Female	2.06 [1.39 to 3.04]	<0.001	1.70 [1.02 to 2.84]	0.04
White Female	1.40 [0.96 to 2.03]	0.08	1.19 [0.77 to 1.84]	0.43
Black Male	2.63 [1.96 to 3.54]	<0.001	2.42 [1.76 to 3.34]	<0.001
Hispanic Male	2.00 [1.27 to 3.15]	0.003	1.81 [1.14 to 2.86]	0.01
White Male	1 [Reference]		1 [Reference]	
18 to 44			1.32 [0.80 to 2.17]	0.27
45 to 54			1.37 [0.96 to 1.95]	0.08
55 to 64			1.39 [1.06 to 1.82]	0.02
65 to 74			1 [Reference]	
75 or older			0.87 [0.68 to 1.13]	0.31
Transportation for basic needs				
Black Female	1.68 [1.20 to 2.34]	0.002	1.37 [0.89 to 2.10]	0.16
Hispanic Female	2.00 [1.35 to 2.95]	<0.001	1.44 [0.83 to 2.49]	0.19
White Female	1.34 [0.91 to 1.97]	0.14	1.11 [0.70 to 1.77]	0.66
Black Male	2.02 [1.47 to 2.79]	<0.001	1.92 [1.37 to 2.70]	<0.001

© 2025 Frank DA et al. *JAMA Health Forum*.

	Unadjusted		Adjusted[a]	
	Prevalence ratio [95% CI]	P-value	Prevalence ratio [95% CI]	P-value
Transportation for basic needs (continued)				
Hispanic Male	1.61 [1.13 to 2.31]	0.009	1.32 [0.85 to 2.06]	0.22
White Male	1 [Reference]		1 [Reference]	
18 to 44			1.49 [0.91 to 2.42]	0.11
45 to 54			0.77 [0.50 to 1.20]	0.25
55 to 64			0.98 [0.73 to 1.33]	0.91
65 to 74			1 [Reference]	
75 or older			0.68 [0.50 to 0.91]	0.010
Adult caregiving for self or others				
Black Female	1.28 [0.97 to 1.71]	0.09	1.33 [0.89 to 1.97]	0.16
Hispanic Female	1.24 [0.89 to 1.73]	0.21	1.30 [0.76 to 2.23]	0.33
White Female	0.97 [0.68 to 1.39]	0.87	1.01 [0.65 to 1.56]	0.96
Black Male	1.61 [1.20 to 2.16]	0.001	1.63 [1.20 to 2.22]	0.002
Hispanic Male	1.22 [0.88 to 1.68]	0.24	1.25 [0.84 to 1.84]	0.27
White Male	1 [Reference]		1 [Reference]	
18 to 44			0.99 [0.53 to 1.86]	0.97
45 to 54			0.85 [0.54 to 1.31]	0.46
55 to 64			1.14 [0.84 to 1.55]	0.39
65 to 74			1 [Reference]	
75 or older			1.11 [0.84 to 1.47]	0.45
Managing experiences of discrimination				
Black Female	4.05 [2.89 to 5.67]	<0.001	2.68 [1.82 to 3.95]	<0.001
Hispanic Female	4.20 [2.83 to 6.24]	<0.001	2.69 [1.68 to 4.29]	<0.001
White Female	2.33 [1.55 to 3.50]	<0.001	1.60 [1.03 to 2.50]	0.04
Black Male	3.33 [2.36 to 4.71]	<0.001	2.73 [1.89 to 3.95]	<0.001
Hispanic Male	1.99 [1.33 to 2.99]	<0.001	1.54 [0.99 to 2.38]	0.06
White Male	1 [Reference]		1 [Reference]	
18 to 44			1.83 [1.21 to 2.78]	0.004
45 to 54			2.16 [1.53 to 3.06]	<0.001
55 to 64			1.77 [1.33 to 2.36]	<0.001
65 to 74			1 [Reference]	
75 or older			0.53 [0.37 to 0.76]	<0.001
Getting additional education or job training				
Black Female	3.05 [2.05 to 4.54]	<0.001	1.45 [0.94 to 2.26]	0.10
Hispanic Female	3.28 [2.10 to 5.13]	<0.001	1.24 [0.74 to 2.07]	0.42
White Female	1.80 [1.11 to 2.94]	0.02	0.93 [0.56 to 1.55]	0.77
Black Male	2.46 [1.62 to 3.74]	<0.001	2.02 [1.32 to 3.09]	0.001
Hispanic Male	2.68 [1.61 to 4.44]	<0.001	1.45 [0.85 to 2.49]	0.17

© 2025 Frank DA et al. *JAMA Health Forum*.

	Unadjusted		Adjusted[a]	
	Prevalence ratio [95% CI]	P-value	Prevalence ratio [95% CI]	P-value
Getting additional education or job training (continued)				
White Male	1 [Reference]		1 [Reference]	
18 to 44			6.40 [4.18 to 9.79]	<0.001
45 to 54			3.65 [2.45 to 5.42]	<0.001
55 to 64			2.68 [1.83 to 3.91]	<0.001
65 to 74			1 [Reference]	
75 or older			0.31 [0.18 to 0.54]	<0.001
Getting or maintaining housing				
Black Female	1.95 [1.39 to 2.74]	<0.001	1.32 [0.89 to 1.96]	0.16
Hispanic Female	2.91 [1.89 to 4.49]	<0.001	1.81 [1.10 to 2.99]	0.02
White Female	1.31 [0.84 to 2.04]	0.23	0.92 [0.57 to 1.50]	0.75
Black Male	2.67 [1.90 to 3.74]	<0.001	2.31 [1.61 to 3.31]	<0.001
Hispanic Male	2.48 [1.54 to 4.00]	<0.001	1.88 [1.18 to 3.02]	0.008
White Male	1 [Reference]		1 [Reference]	
18 to 44			1.81 [1.17 to 2.79]	0.008
45 to 54			1.36 [0.90 to 2.06]	0.15
55 to 64			1.34 [0.97 to 1.84]	0.07
65 to 74			1 [Reference]	
75 or older			0.45 [0.31 to 0.65]	<0.001
Finding or keeping work				
Black Female	2.45 [1.62 to 3.70]	<0.001	1.17 [0.74 to 1.85]	0.51
Hispanic Female	3.19 [2.02 to 5.04]	<0.001	1.21 [0.70 to 2.09]	0.49
White Female	2.02 [1.26 to 3.25]	0.004	1.03 [0.62 to 1.72]	0.90
Black Male	1.64 [1.03 to 2.62]	0.04	1.31 [0.82 to 2.10]	0.26
Hispanic Male	2.09 [1.17 to 3.75]	0.01	1.14 [0.62 to 2.10]	0.66
White Male	1 [Reference]		1 [Reference]	
18 to 44			6.25 [3.70 to 10.56]	<0.001
45 to 54			3.17 [1.96 to 5.12]	<0.001
55 to 64			3.17 [2.02 to 4.98]	<0.001
65 to 74			1 [Reference]	
75 or older			0.21 [0.11 to 0.39]	<0.001
Obtaining childcare				
Black Female	3.99 [1.75 to 9.12]	0.001	2.28 [0.94 to 5.54]	0.07
Hispanic Female	6.41 [2.99 to 13.73]	<0.001	2.78 [1.19 to 6.48]	0.02
White Female	5.65 [2.40 to 13.34]	<0.001	3.37 [1.36 to 8.35]	0.009
Black Male	1.84 [0.79 to 4.30]	0.16	1.63 [0.66 to 4.02]	0.29
Hispanic Male	3.78 [1.63 to 8.80]	0.002	2.30 [0.95 to 5.56]	0.07
White Male	1 [Reference]		1 [Reference]	
18 to 44			3.86 [1.79 to 8.32]	<0.001

© 2025 Frank DA et al. *JAMA Health Forum*.

	Unadjusted		Adjusted[a]	
	**Prevalence ratio [95% CI]**	**P-value**	**Prevalence ratio [95% CI]**	**P-value**
Obtaining childcare (continued)				
45 to 54			1.90 [0.64 to 5.64]	0.25
55 to 64			1.31 [0.52 to 3.30]	0.56
65 to 74			1 [Reference]	
75 or older			0.44 [0.17 to 1.18]	0.10

Abbreviations: CI, Confidence Interval
[a] Models were adjusted for age-groups

© 2025 Frank DA et al. *JAMA Health Forum*.

eTable 3: Race-Ethnicity-Sex Differences in Prevalence and Age-Adjusted Prevalence of Getting Support Among Those Reporting Need

	**Unadjusted**		**Adjusted[a]**	
	**Prevalence ratio [95% CI]**	**P-value**	**Prevalence ratio [95% CI]**	**P-value**
Feeling lonely				
Black Female	0.96 [0.78 to 1.17]	0.69	1.01 [0.80 to 1.27]	0.95
Hispanic Female	0.93 [0.72 to 1.21]	0.60	0.97 [0.71 to 1.33]	0.85
White Female	1.05 [0.84 to 1.31]	0.68	1.10 [0.86 to 1.40]	0.46
Black Male	1.08 [0.88 to 1.31]	0.47	1.08 [0.89 to 1.31]	0.42
Hispanic Male	1.10 [0.87 to 1.39]	0.43	1.10 [0.87 to 1.39]	0.43
White Male	1 [Reference]		1 [Reference]	
18 to 44			0.97 [0.73 to 1.28]	0.82
45 to 54			0.93 [0.74 to 1.18]	0.57
55 to 64			0.95 [0.79 to 1.15]	0.62
65 to 74			1 [Reference]	
75 or older			1.24 [1.05 to 1.46]	0.01
Feeling socially isolated				
Black Female	0.95 [0.77 to 1.17]	0.64	0.99 [0.78 to 1.25]	0.92
Hispanic Female	1.05 [0.83 to 1.33]	0.66	1.11 [0.83 to 1.48]	0.49
White Female	0.91 [0.71 to 1.17]	0.47	0.94 [0.72 to 1.23]	0.66
Black Male	1.07 [0.87 to 1.32]	0.52	1.06 [0.87 to 1.29]	0.56
Hispanic Male	1.06 [0.84 to 1.33]	0.62	1.07 [0.85 to 1.35]	0.56
White Male	1 [Reference]		1 [Reference]	
18 to 44			0.95 [0.70 to 1.28]	0.73
45 to 54			0.98 [0.76 to 1.26]	0.87
55 to 64			1.03 [0.85 to 1.26]	0.74
65 to 74			1 [Reference]	
75 or older			1.24 [1.03 to 1.50]	0.02
Paying for basics				
Black Female	0.82 [0.63 to 1.08]	0.16	0.87 [0.63 to 1.19]	0.39
Hispanic Female	0.90 [0.64 to 1.27]	0.55	0.95 [0.62 to 1.46]	0.83
White Female	0.92 [0.66 to 1.27]	0.60	0.98 [0.69 to 1.37]	0.89
Black Male	0.95 [0.73 to 1.24]	0.71	0.95 [0.74 to 1.21]	0.66
Hispanic Male	1.12 [0.81 to 1.54]	0.49	1.14 [0.83 to 1.56]	0.42
White Male	1 [Reference]		1 [Reference]	
18 to 44			0.85 [0.57 to 1.26]	0.41
45 to 54			0.85 [0.62 to 1.17]	0.33
55 to 64			0.82 [0.64 to 1.04]	0.10
65 to 74			1 [Reference]	
75 or older			1.04 [0.84 to 1.29]	0.69
Paying for food				
Black Female	0.88 [0.69 to 1.13]	0.32	0.90 [0.67 to 1.21]	0.48

© 2025 Frank DA et al. *JAMA Health Forum*.

	Unadjusted		Adjusted[a]	
	Prevalence ratio [95% CI]	P-value	Prevalence ratio [95% CI]	P-value
Paying for food (continued)				
Hispanic Female	1.09 [0.83 to 1.44]	0.53	1.09 [0.75 to 1.58]	0.65
White Female	0.89 [0.64 to 1.22]	0.45	0.91 [0.64 to 1.30]	0.61
Black Male	1.00 [0.78 to 1.28]	0.97	0.99 [0.78 to 1.25]	0.94
Hispanic Male	1.05 [0.80 to 1.37]	0.72	1.04 [0.79 to 1.37]	0.77
White Male	1 [Reference]		1 [Reference]	
18 to 44			0.95 [0.67 to 1.35]	0.77
45 to 54			0.83 [0.61 to 1.14]	0.26
55 to 64			0.86 [0.68 to 1.09]	0.22
65 to 74			1 [Reference]	
75 or older			1.10 [0.89 to 1.36]	0.37
Accessing the internet at home				
Black Female	1.00 [0.79 to 1.27]	>0.99	0.93 [0.69 to 1.26]	0.63
Hispanic Female	0.97 [0.71 to 1.32]	0.86	0.84 [0.56 to 1.26]	0.40
White Female	1.06 [0.82 to 1.36]	0.68	1.03 [0.77 to 1.36]	0.86
Black Male	0.95 [0.77 to 1.16]	0.59	0.96 [0.78 to 1.19]	0.73
Hispanic Male	0.93 [0.71 to 1.22]	0.59	0.85 [0.61 to 1.18]	0.33
White Male	1 [Reference]		1 [Reference]	
18 to 44			1.34 [0.93 to 1.92]	0.11
45 to 54			1.03 [0.70 to 1.51]	0.90
55 to 64			1.11 [0.87 to 1.43]	0.41
65 to 74			1 [Reference]	
75 or older			1.06 [0.82 to 1.36]	0.67
Assistance with legal issues				
Black Female	0.80 [0.56 to 1.15]	0.23	0.84 [0.54 to 1.30]	0.44
Hispanic Female	0.63 [0.38 to 1.04]	0.07	0.60 [0.30 to 1.21]	0.15
White Female	0.77 [0.48 to 1.23]	0.28	0.82 [0.48 to 1.40]	0.46
Black Male	0.94 [0.66 to 1.34]	0.73	0.89 [0.64 to 1.24]	0.48
Hispanic Male	0.62 [0.36 to 1.09]	0.10	0.58 [0.31 to 1.06]	0.08
White Male	1 [Reference]		1 [Reference]	
18 to 44			1.05 [0.53 to 2.06]	0.90
45 to 54			0.64 [0.35 to 1.16]	0.14
55 to 64			0.86 [0.59 to 1.25]	0.41
65 to 74			1 [Reference]	
75 or older			1.32 [0.99 to 1.75]	0.06
Transportation for basic needs				
Black Female	0.93 [0.72 to 1.20]	0.58	0.95 [0.70 to 1.28]	0.73
Hispanic Female	0.86 [0.63 to 1.19]	0.37	0.87 [0.58 to 1.29]	0.48
White Female	0.88 [0.65 to 1.19]	0.41	0.91 [0.66 to 1.26]	0.57
Black Male	0.94 [0.75 to 1.19]	0.62	0.96 [0.77 to 1.20]	0.72

© 2025 Frank DA et al. *JAMA Health Forum*.

	Unadjusted		Adjusted[a]	
	Prevalence ratio [95% CI]	P-value	Prevalence ratio [95% CI]	P-value
Transportation for basic needs (continued)				
Hispanic Male	0.99 [0.76 to 1.30]	0.95	0.98 [0.75 to 1.28]	0.86
White Male	1 [Reference]		1 [Reference]	
18 to 44			1.07 [0.76 to 1.52]	0.69
45 to 54			0.94 [0.66 to 1.36]	0.76
55 to 64			1.02 [0.80 to 1.29]	0.88
65 to 74			1 [Reference]	
75 or older			1.21 [0.99 to 1.47]	0.06
Adult caregiving for self or others				
Black Female	0.91 [0.72 to 1.16]	0.44	1.00 [0.76 to 1.33]	0.97
Hispanic Female	0.85 [0.64 to 1.13]	0.25	0.92 [0.63 to 1.33]	0.65
White Female	0.96 [0.72 to 1.28]	0.79	1.14 [0.91 to 1.43]	0.24
Black Male	0.99 [0.78 to 1.25]	0.92	0.99 [0.80 to 1.22]	0.90
Hispanic Male	1.01 [0.79 to 1.28]	0.94	0.97 [0.76 to 1.23]	0.78
White Male	1 [Reference]		1 [Reference]	
18 to 44			1.03 [0.68 to 1.54]	0.90
45 to 54			0.46 [0.27 to 0.79]	0.005
55 to 64			0.91 [0.70 to 1.19]	0.51
65 to 74			1 [Reference]	
75 or older			1.17 [0.96 to 1.42]	0.12
Managing experiences of discrimination				
Black Female	1.04 [0.62 to 1.75]	0.87	1.16 [0.73 to 1.86]	0.52
Hispanic Female	1.24 [0.70 to 2.18]	0.47	1.65 [1.00 to 2.72]	0.05
White Female	1.08 [0.57 to 2.05]	0.81	1.18 [0.65 to 2.16]	0.58
Black Male	1.38 [0.83 to 2.29]	0.21	1.30 [0.83 to 2.02]	0.25
Hispanic Male	1.20 [0.69 to 2.09]	0.52	1.32 [0.82 to 2.11]	0.25
White Male	1 [Reference]		1 [Reference]	
18 to 44			0.48 [0.28 to 0.83]	0.008
45 to 54			0.84 [0.54 to 1.33]	0.46
55 to 64			0.99 [0.71 to 1.38]	0.96
65 to 74			1 [Reference]	
75 or older			1.19 [0.82 to 1.72]	0.37
Getting additional education or job training				
Black Female	0.84 [0.48 to 1.48]	0.55	0.73 [0.42 to 1.26]	0.26
Hispanic Female	1.12 [0.62 to 2.01]	0.70	0.88 [0.48 to 1.61]	0.68
White Female	1.20 [0.66 to 2.18]	0.56	1.01 [0.56 to 1.84]	0.97
Black Male	0.98 [0.55 to 1.73]	0.94	0.96 [0.55 to 1.68]	0.89
Hispanic Male	0.72 [0.36 to 1.45]	0.36	0.59 [0.28 to 1.24]	0.16

© 2025 Frank DA et al. *JAMA Health Forum*.

	Unadjusted		Adjusted[a]	
	**Prevalence ratio [95% CI]**	**P-value**	**Prevalence ratio [95% CI]**	**P-value**
Getting additional education or job training (continued)				
White Male	1 [Reference]		1 [Reference]	
18 to 44			1.96 [1.15 to 3.36]	0.01
45 to 54			1.40 [0.78 to 2.50]	0.26
55 to 64			1.04 [0.59 to 1.84]	0.88
65 to 74			1 [Reference]	
75 or older			1.24 [0.52 to 2.99]	0.62
Getting or maintaining housing				
Black Female	0.84 [0.59 to 1.20]	0.34	0.83 [0.56 to 1.23]	0.35
Hispanic Female	1.28 [0.92 to 1.79]	0.15	1.23 [0.79 to 1.92]	0.36
White Female	0.71 [0.43 to 1.16]	0.17	0.70 [0.41 to 1.17]	0.17
Black Male	0.98 [0.70 to 1.37]	0.89	0.97 [0.69 to 1.35]	0.85
Hispanic Male	0.78 [0.46 to 1.33]	0.36	0.75 [0.44 to 1.27]	0.28
White Male	1 [Reference]		1 [Reference]	
18 to 44			1.14 [0.71 to 1.81]	0.59
45 to 54			1.05 [0.66 to 1.69]	0.83
55 to 64			1.07 [0.74 to 1.54]	0.72
65 to 74			1 [Reference]	
75 or older			1.28 [0.91 to 1.81]	0.16
Finding or keeping work				
Black Female	0.78 [0.45 to 1.34]	0.37	0.58 [0.35 to 0.94]	0.03
Hispanic Female	1.22 [0.72 to 2.06]	0.47	0.81 [0.51 to 1.30]	0.39
White Female	1.10 [0.63 to 1.93]	0.74	0.83 [0.51 to 1.34]	0.45
Black Male	0.95 [0.52 to 1.72]	0.85	0.82 [0.48 to 1.41]	0.47
Hispanic Male	0.71 [0.35 to 1.47]	0.36	0.49 [0.23 to 1.06]	0.07
White Male	1 [Reference]		1 [Reference]	
18 to 44			1.54 [0.91 to 2.61]	0.11
45 to 54			0.82 [0.42 to 1.60]	0.55
55 to 64			0.64 [0.34 to 1.21]	0.17
65 to 74			1 [Reference]	
75 or older			1.15 [0.52 to 2.54]	0.72
Obtaining childcare				
Black Female	1.11 [0.34 to 3.60]	0.86	1.28 [0.44 to 3.78]	0.65
Hispanic Female	2.21 [0.80 to 6.15]	0.13	2.39 [0.94 to 6.09]	0.07
White Female	1.72 [0.54 to 5.46]	0.35	1.93 [0.70 to 5.37]	0.20
Black Male	2.06 [0.69 to 6.12]	0.19	1.82 [0.71 to 4.72]	0.21
Hispanic Male	1.37 [0.43 to 4.40]	0.60	1.41 [0.49 to 4.07]	0.53
White Male	1 [Reference]		1 [Reference]	
18 to 44			0.58 [0.28 to 1.19]	0.13

© 2025 Frank DA et al. *JAMA Health Forum*.

	Unadjusted		Adjusted[a]	
	**Prevalence ratio [95% CI]**	**P-value**	**Prevalence ratio [95% CI]**	**P-value**
Obtaining childcare (continued)				
45 to 54			0.56 [0.22 to 1.39]	0.21
55 to 64			0.73 [0.32 to 1.63]	0.44
65 to 74			1 [Reference]	
75 or older			0.74 [0.28 to 2.00]	0.55

Abbreviations: CI, Confidence Interval
[a] Models were adjusted for age-groups.

© 2025 Frank DA et al. *JAMA Health Forum*.
