# Supplementary material for: Racial, Ethnic, and Sex Differences in Need and Receipt of Support for Social Needs Among Veterans
Source: JAMA Health Forum. 2025 May 2;6(5):e250992. doi: 10.1001/jamahealthforum.2025.0992 (PMC12048852; doi:10.1001/jamahealthforum.2025.0992)
Supplement: Supplement 2. — Data Sharing Statement [file jamahealthforum-e250992-s002.pdf]

Data Sharing Statement

Frank. Racial, Ethnic, and Sex Differences in Need and Receipt of Support for Social Needs Among Veterans. *JAMA Health Forum*. Published May 02, 2025.
doi:10.1001/jamahealthforum.2025.0992

Data
Data available: No

Additional Information
Explanation for why data not available: We will not make the data available due to VHA data security restrictions.
